# “You are not allowed to break down”: psychological safety and the hidden curriculum of psychiatry training in China

**DOI:** 10.3389/fpsyg.2026.1961068

**Published:** 2026-09-17

**Authors:** Cong Zhou, Xianyang Xie, Aoxue Zhang, Sen Li

**Affiliations:** 1 School of Mental Health, Jining Medical University, Jining, China; 2 Department of Psychology, Affiliated Hospital of Jining Medical University, Jining, China; 3 Department of Psychiatry, Jining Rencheng Mental Hospital, Jining, China; 4 Department of Psychology, Jining Genshine Children’s Rehabilitation Hospital, Jining, China

**Keywords:** ecological approaches, hidden curriculum, medical education, professional identity formation, psychiatry training, psychological safety, trainee mental health

## Abstract

Mental health among medical trainees has typically been framed as an individual deficit, addressed through resilience training and counseling services. This perspective examines how the hidden curriculum within Chinese psychiatry training may shape psychological safety and professional identity formation. Drawing on psychological safety theory, hidden curriculum scholarship, Bronfenbrenner’s ecological systems theory, and recent empirical literature on medical trainee distress in China, we propose a theoretically derived and empirically testable conceptual model through which experiences of vulnerability may become socially and professionally invisible. The proposed vulnerability-to-invisibility process describes four interconnected phases: the encounter with vulnerability, perceived or enacted prohibition of emotional expression, internalization of silence, and potential fragmentation of professional identity. Existing evidence supports several constituent components of this pathway, but direct empirical evidence establishing the complete sequence remains limited. We therefore call for institutional reforms that embed psychological safety into clinical training, including protected reflective spaces, reflective faculty modeling, peer support, and curriculum-integrated mentorship, while emphasizing adaptive emotional regulation rather than forced suppression.

## Introduction

The mental health of medical trainees has become a global concern. Meta-analytic evidence indicates that depression affects approximately 27% of medical students worldwide, with suicidal ideation reported in more than 11% of trainees (Rotenstein et al., 2016). In China, the burden appears equally severe. A meta-analysis of 113 studies involving over 185,000 Chinese university students estimated an overall depression prevalence of 28.4% (Gao et al., 2020). Burnout rates among United States medical students, residents, and early career physicians significantly exceed those of the general population, suggesting that medical training itself exerts pathogenic effects (Dyrbye et al., 2014). In China, a cross-sectional study of 330 residents across four standardized residency training sites in Shanghai reported an overall burnout rate of 71.4%, with occupational stress identified as a significant risk factor and social support as a protective factor (Huang et al., 2020). These figures demand attention not merely as indicators of individual suffering but also as signals that features of medical education environments may contribute to or compound trainee distress. When students experiencing pre-existing or training-related distress enter psychiatry rotations, the training environment may either buffer or compound their vulnerability. Whether and how the hidden curriculum contributes to this process remains an important empirical question. While stress is present across all clinical disciplines, including orthopedics and gynecology, psychiatry presents distinctive stressors that may make psychological safety particularly consequential: intense emotional labor, exposure to patient violence, societal stigma toward both the specialty and its patients, and the expectation that clinicians use their own emotional responses as clinical data. The clerkship student occupies an ambiguous identity position, neither fully student nor physician, as they are simultaneously evaluated on both academic knowledge and clinical performance without clear guidelines for emotional expression. This ambiguity intensifies the psychological impact of the hidden curriculum.

For decades, the dominant response to trainee distress has centered on individual-level solutions: stress management workshops, mindfulness applications, and appeals for greater resilience. While well-intentioned, this approach risks what might be termed the “resilience discourse,” wherein structural problems are reframed as personal inadequacies. When a psychiatry resident experiences emotional exhaustion after months of overnight shifts, traumatic patient encounters, and relentless documentation demands, telling that resident to practice self-care individualizes a problem that is fundamentally institutional.

Importantly, emerging evidence from Chinese psychiatry training provides contextual support for this concern, although it does not directly establish the proposed vulnerability-to-invisibility process. A qualitative study of psychiatry residency training in a tertiary hospital identified limitations in joint training, training formats, and research development, highlighting structural challenges within Chinese psychiatry residency education (Wang et al., 2017). A large nationwide survey of 3,783 psychiatrists and psychiatry residents in China found that fewer than half (47.2%) would choose psychiatry again if given the choice, and that negative professional identity, burnout, and job dissatisfaction were associated with reluctance to reselect psychiatry (Gu et al., 2023). In addition, research on Chinese psychiatry residents suggests that structured reflective interventions such as Balint groups may improve psychological wellbeing and reduce dimensions of burnout (Wang et al., 2024). These studies do not demonstrate that emotional vulnerability is systematically suppressed or that suppression causes identity fragmentation. Rather, they establish the broader contextual plausibility and clinical importance of the problem addressed by the present conceptual model.

This perspective proposes an alternative framing. In the Chinese medical education context, psychiatry training in China encompasses interconnected undergraduate and postgraduate stages, which share institutional and cultural environments but involve different roles, responsibilities, and stressors. For medical students majoring in clinical medicine, the psychiatry clerkship comprises mandatory 4–8-week clinical rotations during Years 4 to 5 of the 5-year undergraduate curriculum. For medical students majoring in psychiatry, the clerkship is substantially longer, with more course hours and greater clinical exposure than the standard clinical medicine curriculum (Jing et al., 2018). In both undergraduate forms, trainees observe patient care, assist in documentation, and participate in risk assessments under direct supervision without independent clinical responsibility. These undergraduate clerkships are longstanding components of the Chinese medical curriculum, not created by the 2014 reform which targeted postgraduate training only. Selection is by curricular assignment rather than student choice; all students in the respective majors are assigned to these rotations as compulsory, non-elective requirements. The postgraduate residency training system, launched in 2014 and standardized nationwide by 2020, spans 33 months for medical graduates and involves progressive clinical independence (Liu et al., 2025). While this perspective focuses primarily on the undergraduate clerkship, the three stages share overlapping institutional cultures and hidden curricula that suppress emotional vulnerability. We argue that trainee mental health in Chinese psychiatry clerkship must be understood through two interconnected lenses: psychological safety and the hidden curriculum. Psychological safety, defined by Edmondson as the shared belief that a team is safe for interpersonal risk-taking without fear of humiliation or retaliation (Edmondson, 1999), has been identified as a critical precondition for learning in medical education (Tsuei et al., 2019). The hidden curriculum, conceptualized by Hafferty as the set of influences that function at the level of organizational structure and culture to impact learning (Hafferty and Franks, 1994; Hafferty, 1998), encompasses the unwritten rules, power dynamics, and ritualized practices that shape how trainees become professionals. When these two constructs intersect in the context of Chinese psychiatry training, they produce a learning environment in which emotional vulnerability is systematically silenced and professional identity formation is compromised. The vulnerability-to-invisibility process may therefore operate differently across training stages. Undergraduate trainees may be particularly vulnerable to uncertainty surrounding transitional professional identity, limited clinical authority, and unfamiliarity with psychiatric practice. Residents, by contrast, assume progressively greater clinical responsibility and may experience additional pressures related to workload, competence, evaluation, and career uncertainty. The same hidden curriculum may consequently be expressed differently: undergraduate trainees may learn that emotional disclosure is inconsistent with being a competent future physician, whereas residents may experience disclosure as potentially threatening their professional standing or perceived competence. Importantly, the 2014 reform substantially standardized postgraduate training but did not itself create the undergraduate psychiatry clerkship. Whether the reform has altered the hidden curriculum surrounding emotional expression and psychological safety remains unclear and warrants empirical investigation.

Our argument unfolds in four stages. First, we conceptualize psychological safety and the hidden curriculum as analytical tools for understanding trainee experience. Second, we examine the distinctive features of psychiatry training in China that render trainees particularly vulnerable to low psychological safety. Third, we elaborate the “vulnerability-to-invisibility” process, tracing how unsupported vulnerability is transformed into silenced distress and fragmented identity. Finally, we outline an ecological reframing that shifts responsibility from the individual trainee to the institutional systems of teaching, mentorship, and clinical governance.

## Conceptualizing psychological safety and the hidden curriculum

The concept of psychological safety was introduced by Edmondson (1999) as a shared belief within a group that it is safe to take interpersonal risks. Edmondson (1999) defined psychological safety as “a sense of confidence that the team will not embarrass, reject or punish someone for speaking up”. In her foundational study, Edmondson (1999) demonstrated that teams with higher psychological safety engage in more learning behaviors, which in turn leads to better performance. The construct has since been applied extensively in organizational behavior and, more recently, in medical education (Tsuei et al., 2019).

Although psychological safety was initially developed within Western organizational research, its central proposition—that perceived interpersonal risk shapes whether individuals speak up, ask questions, disclose uncertainty, and engage in learning—is potentially applicable across educational and clinical settings. However, its expression may differ across cultural and hierarchical contexts. In Chinese medical training, concerns about face, status, relational trust, and hierarchical evaluation may influence how trainees interpret the interpersonal risks of disclosure. Psychological safety in this context may therefore require particular attention to vertical as well as horizontal relationships: trainees need confidence not only that peers will respond supportively, but also that supervisors will not interpret disclosure as incompetence, loss of face, or lack of professional commitment.

In health professions education, psychological safety has been recognized as essential to creating positive learning environments. Clark’s four-stage model of psychological safety (Inclusion Safety, Learner Safety, Contributor Safety, and Challenger Safety) has been applied to various educational settings including clinical training (Dong et al., 2025). Learner Safety, the stage most relevant to trainee experience, refers to feeling comfortable asking questions, exploring new topics, and learning from mistakes (Dong et al., 2025). When Learner Safety is absent, trainees avoid seeking clarification, hide uncertainties, and refrain from admitting errors, all of which are essential to professional growth.

The hidden curriculum, a concept first introduced by Philip Jackson in 1968 and later applied to medical education by Hafferty (1998), refers to the implicit lessons, values, and norms transmitted outside the formal curriculum. Hafferty argued that beyond formal curricular reform, the organizational culture, policies, evaluation systems, and everyday practices of medical institutions powerfully shape professional identity formation in trainees (Hafferty and Franks, 1994; Hafferty, 1998). He distinguished among three interrelated components of medical training: the formal curriculum, the informal curriculum, and the hidden curriculum (Hafferty, 1998). The hidden curriculum is not merely an adjunct to formal education; it is often more influential in shaping professional attitudes and behaviors than explicitly taught content.

The hidden curriculum in medical education has been shown to include stressful work environments, unempathic role models, and the prioritization of biomedical knowledge over humanistic values (Neumann et al., 2011). It can provoke anxiety and cause medical students to adapt by becoming cynical, distanced, and less empathic (Neumann et al., 2011). More recently, scholars have examined how the hidden curriculum reinforces implicit biases, shapes professional identity formation, and influences trainee wellbeing (Torralba et al., 2020). The intersection of psychological safety and the hidden curriculum is particularly significant (Torralba et al., 2020). When the hidden curriculum communicates that vulnerability is unacceptable, it directly undermines psychological safety. Trainees learn not through formal instruction but through observation and experience that certain expressions of distress are risky, that certain questions are unwelcome, and that certain emotions are unprofessional. These implicit lessons are often more powerful than explicit messages about wellbeing or self-care.

## Psychiatry training in China: a distinctive context

While this perspective focuses primarily on undergraduate clerkships, understanding the broader training ecology requires appreciating how the hidden curriculum operates across all stages. The following description of the postgraduate residency system illustrates the institutional context into which undergraduate trainees will eventually graduate, and the shared cultural norms that permeate both stages. To understand how the hidden curriculum operates in Chinese psychiatry training, it is necessary to appreciate the structural and cultural context in which it unfolds.

China’s standardized residency training system, launched in 2014 by the National Health Commission and implemented nationwide by 2020, requires medical graduates to complete at least 33 months of residency training. For psychiatry, the training program consists of 9 months of rotations in internal medicine, 18 months of clinical rotations in psychiatric inpatient services (including 12 months in acute psychiatric wards and 6 months in low-acuity or rehabilitation wards), 3 months in psychiatric outpatient clinics or emergency units, and 3 months of elective rotations.

Psychiatry trainees in China face distinctive challenges. They work in a specialty that remains heavily stigmatized within both the medical profession and society at large. They confront patients with severe mental illness, often in under-resourced settings where the ratio of staff to patients is far from optimal. They are exposed to the risk of patient violence. They navigate the emotional demands of therapeutic relationships with patients who may be suicidal, psychotic, or profoundly distressed, demands for which formal training provides little preparation.

Yet the emotional responses these experiences evoke, including fear, helplessness, grief, and exhaustion, are rarely acknowledged within the training environment. Instead, trainees encounter a pervasive implicit message: that “good” psychiatrists are those who remain unflappable, who do not flinch in the face of psychosis, and who do not admit to being afraid. This message is transmitted through multiple channels: through senior clinicians who model emotional detachment as professionalism, through evaluation systems that reward stoicism, through peer cultures that stigmatize expressions of distress, and through institutional practices that prioritize throughput over reflection.

The result is a profound mismatch between what trainees experience and what they are permitted to express. This mismatch is not incidental to psychiatric training; it is systematically produced by the hidden curriculum. Trainees learn that to admit fear is to admit inadequacy, to express sadness is to lack resilience, and to acknowledge uncertainty is to reveal incompetence. They learn that the professional self is one that does not “break down.”

Empirical research on Chinese college students has documented that self-stigma of seeking psychological help functions as one of the most significant barriers to accessing care (Vogel et al., 2006). Among medical students, this stigma is compounded by the fear that disclosure of psychological distress will jeopardize career advancement, licensing, or standing within training programs (Dyrbye et al., 2015). A large-sample nationwide survey of 3,783 psychiatrists and psychiatry residents in China found that less than half (47.2%) would choose psychiatry again if given the choice, and one-quarter would definitely not (Gu et al., 2023). Those who would not choose psychiatry again were significantly more likely to have a negative professional identity, experience job burnout, and be dissatisfied with their work (Gu et al., 2023).

Moreover, the hidden curriculum in Chinese psychiatry training is reinforced by structural features of the healthcare system. When trainees feel they lack adequate psychological support, they become emotionally drained and subsequently detach from their work or struggle to empathize with patients (Neumann et al., 2011). The coping strategies they adopt are shaped by the very environments that deny them support.

## From vulnerability to invisibility: the mechanism of identity erosion

We propose that the hidden curriculum of emotional prohibition in Chinese psychiatry training operates through a specific mechanism: the transformation of vulnerability into invisibility. This process unfolds through four interconnected phases.

### Phase one: the encounter with vulnerability

Psychiatry trainees may encounter substantial sources of vulnerability from early in clinical training. They confront patients whose suffering is visible and overwhelming. They witness psychotic breaks, suicide attempts, and severe self-harm. They are asked to conduct risk assessments, to make clinical judgments under uncertainty, and to contain the emotional intensity of patients in crisis. They are exposed to the possibility, sometimes the reality, of patient violence. These experiences may evoke legitimate emotional responses: fear, anxiety, sadness, and helplessness.

These responses are not signs of weakness or unsuitability for the profession. They are normal human reactions to emotionally demanding situations. Indeed, the capacity to experience and reflect on these emotions may be essential to developing the empathy and clinical judgment that characterize excellent psychiatric practice. Yet the hidden curriculum communicates otherwise.

### Phase two: the prohibition of expression

When trainees attempt to express these emotions, whether admitting fear after a violent patient encounter, acknowledging diagnostic uncertainty, or sharing exhaustion, they encounter implicit or explicit prohibition. This prohibition takes many forms: a senior clinician’s dismissive response, a peer’s uncomfortable silence, an evaluation comment that questions their “fitness” for the specialty, or an institutional culture that simply does not provide spaces for such conversations. Peer culture may function as both a transmission pathway and a potential protective factor. Senior trainees may socialize junior colleagues through informal conversations, advice, jokes, and everyday demonstrations of what is considered acceptable professional behavior. When stoicism is normalized within the peer group, junior trainees may learn that emotional disclosure is undesirable even without explicit instruction from faculty. Conversely, peer support and peer mentorship may provide a relatively lower-risk context for discussing difficult experiences. Peer relationships should therefore be understood not only as a source through which the hidden curriculum is reproduced, but also as a potential leverage point for intervention.

The prohibition is rarely stated as “you are not allowed to break down.” It is communicated through what is not said, what is not asked, and what is not provided. It is transmitted through role modeling when senior clinicians present themselves as unflappable. It is reinforced through evaluation when emotional expression is interpreted as lack of professionalism. It is embedded in institutional practices when there are no structured opportunities for reflection on emotional experience.

This expectation of composure should not be attributed to a single cultural tradition or treated as a fixed characteristic of Chinese medical culture. Historical Confucian ideals have emphasized self-discipline, relational harmony, role responsibility, and the maintenance of appropriate conduct, and these values can be interpreted in different ways within contemporary medical settings. On one hand, traditions of emotional restraint and hierarchical propriety may make disclosure of uncertainty or distress feel socially risky, particularly when trainees are concerned about losing face or appearing insufficiently competent. On the other hand, Confucian values such as benevolence (ren), responsibility toward others, relational harmony, and self-cultivation may provide cultural resources for supportive mentorship, reflective practice, and compassionate care. The relevant issue is therefore not “Confucianism” itself, but how particular interpretations of emotional restraint and hierarchical professionalism interact with contemporary institutional practices. Moreover, Chinese medical culture is not monolithic. Generational differences, institutional cultures, professional roles, and increasing mental-health awareness may shape attitudes toward emotional expression, and younger clinicians may be more receptive to psychological support and reflective practices.

From this perspective, the concepts of face and relational trust may complement psychological safety theory by helping explain why the interpersonal costs of disclosure can be particularly salient in hierarchical training relationships.

### Phase three: the internalization of silence

If such prohibitions are repeated, trainees may internalize the message that some emotional responses are unacceptable. They may gradually become less willing to disclose fear, uncertainty, sadness, or exhaustion. Over time, some trainees may develop a “professional facade” that conforms to perceived expectations while concealing aspects of their emotional experience.

This internalization is not merely a matter of suppressing emotion. It involves a fundamental reorganization of the trainee’s relationship to their own experience. Emotions that cannot be expressed become emotions that cannot be fully acknowledged, even to oneself. The trainee begins to doubt the legitimacy of their own responses. They may interpret their fear as evidence of inadequacy, their sadness as a character flaw, and their exhaustion as a failure of resilience. They may come to believe that if they were truly suited to psychiatry, they would not feel this way.

### Phase four: the fragmentation of identity

If sustained over time, this process may contribute to fragmentation of professional identity. Professional identity formation (PIF), understood as a process of psychological development and socialization within the profession, requires integration, the capacity to bring one’s values, emotions, and experiences into a coherent sense of professional self (Jarvis-Selinger et al., 2012; Cruess et al., 2014, 2015). When trainees are forced to split off and silence essential aspects of their experience, this integration is undermined (Cruess et al., 2014).

The result is not the formation of a robust professional identity but its erosion. Trainees may develop what appears to be a professional self on the surface, competent, composed, and unflappable, while their internal experience remains fragmented and disconnected. They may lose touch with the emotional responses that could have informed their clinical judgment and enriched their therapeutic relationships. They may become cynical, distanced, and less empathic (Neumann et al., 2011). They may question whether they belong in psychiatry at all.

This fragmentation has consequences that extend beyond training. The nationwide survey by Gu et al. (2023) provides an important contextual indicator: negative professional identity and burnout were associated with reluctance to choose psychiatry again. These findings are consistent with the broader concern that professional identity and wellbeing are important determinants of career attitudes, but they do not establish that the proposed vulnerability-to-invisibility process causes these outcomes. Those who leave the specialty, or who stay but remain disengaged, represent not only a loss to the profession but, in many cases, a consequence of training environments that failed to support authentic professional identity formation. We acknowledge that workforce attrition is multiply determined: economic factors, specialty prestige, geographic distribution of posts, and personal life circumstances all play roles. However, the finding that negative professional identity and burnout are significantly associated with the intention to leave suggests that the quality of the training environment is a modifiable and clinically relevant contributor.

## Toward an ecological reframing: from individual resilience to institutional responsibility

If the hidden curriculum of silence and the absence of psychological safety are structural problems, then their solutions must also be structural. Bronfenbrenner’s ecological systems theory offers a useful framework for this reframing (Jones et al., 2020). Rather than locating the problem within the individual trainee, an ecological approach examines the nested systems that shape trainee experience: the microsystem of immediate clinical relationships, the mesosystem of connections between training and support services, the exosystem of institutional policies, and the macrosystem of cultural values and professional norms (Jones et al., 2020). Each of these levels presents opportunities for intervention.

### Microsystem

At the microsystem level, faculty behavior is paramount. McClintock et al. (2022) emphasize that psychological safety is set by leaders and felt by learners. When faculty members model vulnerability by acknowledging their own uncertainties, discussing their own emotional responses to difficult cases, or admitting when they have made mistakes, they signal to trainees that emotional honesty is compatible with professional competence (McClintock et al., 2022). This is not about lowering standards; it is about creating the conditions under which high standards can be met without sacrificing wellbeing. In practice, faculty vulnerability modeling could be incorporated into existing faculty-development programs rather than introduced as a separate wellness initiative. Short workshops could train supervisors to discuss clinical uncertainty, difficult emotional reactions, and mistakes using a structured format: acknowledge the experience, reflect on its clinical meaning, describe how it was regulated, and identify what was learned. This approach emphasizes reflective professional practice rather than personal disclosure. Faculty should not be required to disclose highly personal information. To reduce resistance, participation could initially be voluntary and subsequently incorporated into teaching-development or supervisory evaluation frameworks where appropriate. In psychiatry training, where faculty are expected to teach therapeutic presence, this modeling is doubly important. A supervisor who discusses their own countertransference reactions demonstrates that emotional engagement is not a liability but a clinical tool.

### Mesosystem

At the mesosystem level, training programs should create protected spaces where trainees can reflect on their experiences without performance pressure. A feasible model in the Chinese context could consist of a monthly 60–90 min small-group reflective session facilitated by a trained psychiatrist, psychologist, or faculty member who is not directly responsible for participants’ performance evaluation. Sessions could combine anonymous written reflection, brief case-based narrative sharing, and voluntary discussion. For trainees who are initially uncomfortable with direct emotional disclosure, written reflection could precede oral discussion. Balint groups and narrative medicine workshops provide possible formats, but the central design principle is that participation should be non-evaluative and confidentiality boundaries should be explicitly explained. These sessions should be scheduled within existing protected educational hours rather than added as unpaid after-hours activities. A qualitative study of psychiatric trainees in Thailand found that a monthly “Exhibition of Failure” reflective session created a safe environment for discussing setbacks and supported connection, self-acceptance, and peer acceptance (Anantapong et al., 2026), illustrating the potential value of structured reflective spaces in psychiatry training.

### Exosystem

At the exosystem level, institutional policies must be revised to remove the punitive consequences of distress disclosure. This includes ensuring that mental health service utilization is not documented in trainee records that are accessible to promotion committees, creating clear protocols for temporary workload reduction when trainees experience psychological crises, and training program directors to respond to distress with support rather than surveillance. Dyrbye et al. (2015) found that medical students with burnout who perceived high levels of stigma were significantly less likely to seek help, and that personal experiences of negative consequences following disclosure reinforced this reluctance. Institutions must therefore audit their own policies to identify and eliminate the sources of this stigma.

### Macrosystem

At the macrosystem level, the goal should not be to reject cultural traditions but to reinterpret professionalism in ways that support both emotional regulation and psychological safety. The legacy of Confucianism in contemporary Chinese medical culture is complex and contested rather than uniform. Confucian traditions have historically emphasized self-discipline, emotional restraint, respect for hierarchy, relational harmony, responsibility toward others, benevolence, and self-cultivation. Some interpretations of these values may reinforce expectations that clinicians remain composed, defer to senior colleagues, and avoid expressions of distress that could be perceived as disruptive or professionally inappropriate. However, the same traditions can also be interpreted as resources for compassionate care, relational responsibility, reflective self-cultivation, and supportive mentorship. The contemporary significance of these values therefore depends on how they are interpreted and enacted within specific institutional and generational contexts.

Importantly, contemporary Chinese medical trainees should not be assumed to share a uniform attitude toward emotional expression or psychological help-seeking. Recent studies of Chinese medical students have identified substantial variation in attitudes toward professional psychological help-seeking, with mental health status, psychological knowledge, family functioning, perceived utility of help-seeking, and stigma all influencing these attitudes (Qiu et al., 2024a,b). Qualitative evidence from Chinese medical graduate students further suggests that interpersonal and hierarchical relationships can shape trainees’ experiences of stress and communication. In one recent study, students described concerns about communicating with supervisors within hierarchical relationships and expressed a preference for more interactive and individualized forms of mental health support (Zhang et al., 2024). These findings suggest that emotional disclosure and help-seeking in Chinese medical education are shaped by multiple interacting individual, interpersonal, and institutional factors rather than by cultural tradition alone.

Generational change may further contribute to this heterogeneity. Younger clinicians and trainees may encounter increasingly visible discussions of mental health and professional wellbeing and may therefore be more receptive to psychological support than some previous generations. However, this possibility should not be interpreted as a simple transition from “traditional” to “modern” values, nor should it be assumed that younger clinicians are no longer influenced by hierarchical expectations. Rather, contemporary trainees remain embedded in institutional structures in which seniority, evaluation, and professional reputation may continue to shape the perceived risks of disclosure. Generational change is therefore better understood as an ongoing negotiation between inherited norms and emerging professional identities, a proposition that warrants further empirical investigation.

The relevant issue is therefore not “Confucianism” itself, but how particular interpretations of emotional restraint, relational obligation, and hierarchical professionalism interact with contemporary institutional practices. Cultural traditions should be understood as dynamic resources that can be selectively reinforced, challenged, or reinterpreted rather than as fixed determinants of trainee behavior. From this perspective, relational harmony and self-cultivation need not imply silence or emotional suppression; they can instead be reframed to support respectful communication, reflective practice, and psychologically safe mentorship. The key distinction is between adaptive composure, which supports clinical functioning, and compelled silence, which prevents trainees from acknowledging experiences that may require reflection or support.

## Distinguishing emotional regulation from emotional suppression

An important conceptual distinction is between emotional regulation and emotional suppression. Emotional regulation refers to recognizing, reflecting upon, and modulating affective responses in accordance with clinical goals and professional boundaries. Emotional suppression, by contrast, involves concealing or denying emotional experiences because disclosure is perceived as unsafe, unacceptable, or professionally damaging. Psychiatry training should cultivate the former rather than inadvertently rewarding the latter. Psychological safety does not imply unrestricted emotional expression; rather, it creates conditions in which trainees can acknowledge difficult experiences, reflect on them, and learn how to regulate them professionally.

This distinction does not imply that all emotions should be expressed openly in clinical settings. Professionalism in psychiatry, as in all medical specialties, requires emotional regulation: the capacity to modulate one’s affective responses to maintain therapeutic boundaries, ensure patient safety, and sustain clinical judgment under pressure. The concern arises when professionalism becomes equated with the absence of emotional experience itself, rather than with the capacity to recognize, reflect upon, and appropriately regulate such experiences. When the hidden curriculum inadvertently rewards suppression, trainees may learn to conceal distress rather than process it reflectively. A psychologically safe training culture would therefore not seek to produce emotionally unregulated clinicians; rather, it would help trainees develop the reflective capacity to recognize, regulate, and appropriately integrate emotional responses into clinical practice.

## Limitations and directions for empirical investigation

Several limitations should be acknowledged. First, the vulnerability-to-invisibility process proposed here is a conceptual model rather than a mechanism established by direct empirical testing. Existing Chinese literature provides evidence of training-related stress, burnout, professional identity concerns, and structural challenges, but does not yet demonstrate that vulnerability progresses through the proposed four phases in a fixed sequence. The model should therefore be regarded as a theoretically derived and empirically testable proposition.

Second, the process may not operate uniformly across trainees or training stages. Generational differences, institutional culture, supervisory style, prior clinical experience, specialty exposure, and individual coping resources may shape how vulnerability is perceived and expressed. The framework should therefore not be interpreted as a deterministic account of Chinese psychiatry training.

Future research should directly investigate the model using qualitative interviews, narrative inquiry, longitudinal cohort studies, and mixed-methods designs. Qualitative studies could examine how trainees describe emotionally difficult clinical encounters, whether and why they disclose these experiences, how supervisors and peers respond, and how these interactions influence professional identity. Quantitative studies could examine whether psychological safety and emotional suppression mediate associations between training stressors and outcomes such as burnout, professional identity, help-seeking, and career intention. Such research would clarify whether the proposed vulnerability-to-invisibility sequence represents a reproducible process, a context-dependent pattern, or a useful conceptual metaphor.

## Conclusion

The mental health challenges faced by medical trainees in China are unlikely to be resolved by resilience workshops or mindfulness apps alone. While individual coping strategies play an important role, training environments also shape whether trainees feel able to acknowledge uncertainty, seek support, and integrate difficult experiences into professional identity. In Chinese psychiatry training, the hidden curriculum may contribute to an environment in which emotional vulnerability is perceived as professionally risky, but the extent and consequences of this process require further empirical investigation.

This perspective has argued for an ecological reframing that locates responsibility for trainee wellbeing not only in the individual but also in the institutional systems of medical education. We do not claim that the hidden curriculum is the sole determinant of trainee distress or workforce attrition. Rather, we propose that it is a potentially modifiable contributor that warrants greater empirical attention in the Chinese context. By strengthening psychological safety, modeling reflective professional practice, creating protected reflective spaces, supporting peer relationships, and reviewing policies that may inadvertently penalize distress disclosure, training programs may create conditions in which trainees can acknowledge vulnerability without sacrificing professional standards. The goal is not to produce trainees who can endure more suffering, but to develop training environments in which difficult experiences can be acknowledged, reflected upon, and transformed into clinically meaningful learning.

It is time for medical educators to answer the hidden curriculum’s implicit voice with a different message: “You are allowed to struggle, and you will be supported when you do.” Only then can psychiatry training fulfill its promise of producing not merely competent technicians but healers who have learned to care for others by first learning to care for themselves.

## Data Availability

The original contributions presented in the study are included in the article/supplementary material, further inquiries can be directed to the corresponding authors.
